# 522. Real-World, Retrospective Descriptive Study of COVID-19 Outpatient Therapeutic Uptake in High-Risk Patients Subsequently Hospitalized for COVID-19 During the Omicron Period in the US

**DOI:** 10.1093/ofid/ofad500.591

**Published:** 2023-11-27

**Authors:** Amie Scott, Laura A Puzniak, Michael V Murphy, Darrin W Benjumea, Andrew Rava, Michael Benigno, Kristen E Allen, Richard Stanford, Fadi Manuel, Ashley S Cha-Silva, Lili Jiang, Maya Reimbaeva, Florin Draica

**Affiliations:** Pfizer, Inc, New York, New York; Pfizer Inc., Collegeville, Pennsylvania; Genesis Research, Hoboken, New Jersey; Genesis Research, LLC, Hoboken, New Jersey; Genesis Research, Hoboken, New Jersey; Pfizer, Inc, New York, New York; Pfizer, Inc., Minneapolis, Minnesota; AESARA, Chapel Hill, North Carolina; AESARA, Chapel Hill, North Carolina; Pfizer, Trumbull, Connecticut; Pfizer, Trumbull, Connecticut; Pfizer, Inc., Minneapolis, Minnesota; Pfizer, Inc., Minneapolis, Minnesota

## Abstract

**Background:**

Since late December 2021, the COVID-19 Omicron variant has been the predominant variant in the US. While considered less severe than previous variants, the burden of severe COVID-19 remains significant, especially in patients with high-risk underlying conditions. Underutilization of outpatient therapeutics has been reported in this population. The objective of this study was to assess the uptake of outpatient COVID-19 therapeutics 30 days prior to COVID-19 hospitalization during the Omicron period, among those high-risk for hospitalization based on CDC-defined criteria.

**Methods:**

Patients with ≥1 inpatient hospitalization, a primary diagnosis of COVID-19 (ICD10 U07.1) and ≥ 1 CDC-defined high-risk condition were identified using Optum's de-Identified Clinformatics® Data Mart Database Date of Death claims data from 01/01/22-11/30/22. Death within 1 calendar month after hospitalization was used as a proxy for inpatient death, due to database limitations. Receipt of a COVID-19 outpatient therapeutic (**Table 1**) in the 30 days prior to hospital admission was assessed by death, age, number of high-risk conditions, and immunocompromised status.

**Figure:**
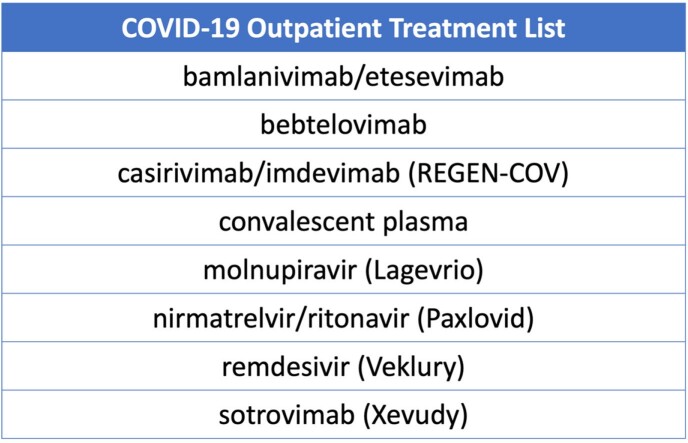

**Results:**

42,939 patients met inclusion criteria, of which 7,209 died within 1 calendar month of hospitalization. At admission, patients were on average 75.7 years old (SD 12.0) and mostly female (51.9%). Patients with death within 1 calendar month were older (79.7 vs. 74.9 years) and more often male (54.1% vs. 46.9%) than those without. Patients were majority white (68.2%), regardless of death. The most common high-risk conditions at hospitalization were hypertension (88.2%), advanced age ( ≥ 65; 86.0%), and heart conditions (78.4%). A higher proportion of the most common conditions were observed in hospitalizations with death within 1 calendar month. Overall, 40,463 (94.2%) of high-risk patients had not received COVID-19 outpatient treatment within 30 days prior to hospitalization, of which 6,886 (17.0%) died within 1 calendar month. **Figure 1** shows key high-risk stratifications.

Percentage of patients treated with COVID-19 outpatient treatments within 30-days prior to hospitalization by key high-risk stratifications

**Figure:**
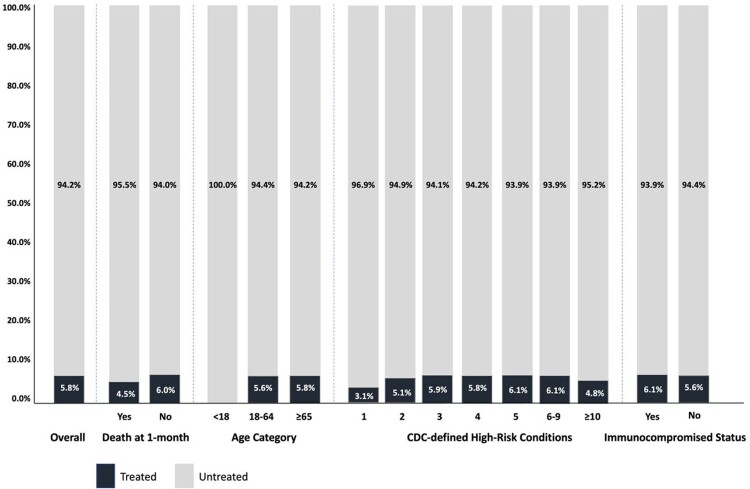

**Conclusion:**

There was low uptake of COVID-19 outpatient treatment pre-hospitalization in patients high-risk for COVID-19 hospitalization, highlighting the persistent burden of hospitalization and death during the Omicron period among this population.

**Disclosures:**

**Amie Scott, MPH**, Pfizer, Inc: Employee|Pfizer, Inc: Stocks/Bonds **Laura A. Puzniak, PhD. MPH**, Pfizer, Inc.: Employee|Pfizer, Inc.: Stocks/Bonds **Michael V. Murphy, BA**, Bellus Health: Advisor/Consultant|Boston Scientific: Employee|Boston Scientific: Stocks/Bonds|EMD Serono: Advisor/Consultant|Pfizer: Advisor/Consultant|Sanofi: Advisor/Consultant|Travere Therapeutics: Advisor/Consultant **Darrin W. Benjumea, MPH**, Akebia Therapeutics: Advisor/Consultant|Arvinas: Advisor/Consultant|Bellerophon Therapeutics: Stocks/Bonds|Bellus Health: Advisor/Consultant|Genesis Research, LLC: Employee|Novartis: Stocks/Bonds|Pfizer Inc.: Stocks/Bonds|Regeneron: Advisor/Consultant|Regeneron: Stocks/Bonds **Andrew Rava, MPH**, AbbVie: Advisor/Consultant|Janssen: Advisor/Consultant|Novartis: Advisor/Consultant|Pfizer, Inc.: Advisor/Consultant|Travere Therapeutics, Inc.: Advisor/Consultant **Michael Benigno, MA**, Pfizer: Employee|Pfizer: Stocks/Bonds **Kristen E. Allen, MPH**, Pfizer, Inc.: Employee|Pfizer, Inc.: Stocks/Bonds **Richard Stanford, PharmD, MS**, Pfizer: Advisor/Consultant **Fadi Manuel, PharmD**, Pfizer: Medical Writing **Ashley S. Cha-Silva, PharmD, MS**, Pfizer Inc.: Employee|Pfizer Inc.: Stocks/Bonds **Maya Reimbaeva, MS**, Pfizer, Inc.: Employee|Pfizer, Inc.: Stocks/Bonds **Florin Draica, MD**, Pfizer Inc.: Honoraria|Pfizer Inc.: Stocks/Bonds

